# Book Reviews

**Published:** 1830-10

**Authors:** 


### CRITICAL ANALYSES.

Quæ laudanda forent, et quæ culpanda, vicissim

Illa, prius, cretâ; mox hæc, carbone, *notamus.*—Persius.

*A Treatise on the Venereal Diseases of the Eye.* By William Lawrence, F.R.S.; late Professor of Anatomy and Surgery to the Royal College of Surgeons in London; Surgeon to St. Bartholomew's Hospital, and Lecturer on Surgery at that Hospital; Surgeon to Bridewell and Bethlehem Hospitals; consulting Surgeon to the London Fever Hospital, and late Surgeon to the London Ophthalmic Infirmary, &c.—8vo. pp. 337. Wilson, 1830.

When we consider the curious and intricate structure of the eye, and the precious sense of which it is the organ, we cannot wonder that philosophers and physiologists in every age have made its anatomy and function their especial objects of investigation. The various diseases to which the eye is subject were formerly less industriously studied by surgeons, and, as the work now before us will show, neither the pathology nor treatment of some of its most serious maladies have, until very recently, been thoroughly understood. The cause is obvious why this particular department of the science of surgery has been less rapidly improved than many others of minor importance. Until within the last few years, the study of ophthalmic surgery was entirely neglected in this country; and hence arose the custom for persons labouring under diseases of the eye to resort to a distinct class of practitioners, whose study and practice did not embrace diseases generally. Surgeons themselves, indeed, conscious of their own ignorance, consigned their patients to such practitioners when they were affected with diseases of the eye. We have no longer to lament such discreditable indolence. English surgeons of extensive experience and undoubted ability have recently done much to improve ophthalmic surgery, and, by the delivery of public lectures expressly upon the subject, the rising members of the profession have been stimulated to study diseases of the eye, which once were considered to form no part of the education of a surgeon, with as much zeal as other branches of their art.

Venereal diseases of the eye, to which Mr. LAWRENCE now exclusively directs our attention, are in many respects peculiarly important, but until the appearance of the present treatise, they had not been professedly or generally considered. The account which Mr. Lawrence gives us of the nature, symptoms, and treatment of the venereal diseases affecting the eye, has been drawn up entirely from his own experience, and numerous cases are circumstantially detailed to show the foundation upon which his opinions rest.

The numerous dissimilar, and for the greater part highly organized, structures of the eye, are not only liable to common disease, but also exhibit peculiar modifications of morbid affection in scrofulous, gouty, and rheumatic constitutions. The eye also suffers from many diseases of the skin: it is liable to cancer, fungus hæmatodes, and melanosis; and, as we might expect, it is exposed to various affections depending immediately and obviously on those morbid poisons, gonorrhœa and syphilis. By various writers such diseases have been mentioned, but by none have they been treated in a satisfactory manner. Mr. HUNTER and Mr. JOHN PEARSON seem hardly to have known that the eye was subject to any affections of a venereal character. The former writer does not treat of gonorrhœal ophthalmia in his work on the Venereal Disease, and he alludes to what he calls *supposed* venereal inflammation of the eyes, merely for the purpose of expressing his doubts whether such cases exist. Mr. Pearson says, in a letter to Mr. Briggs, " I cannot help entertaining some doubts of the propriety of assigning the gonorrhœa as a cause of ophthalmia; since, during a pretty extensive experience of twenty-five years, I have never seen one single instance of inflammation of the eyes which was evidently derived from a gonorrhœa. Mr. Pearson was also of opinion that there are no specific characters by which diseases of the eye or eyelids, produced by the action of the venereal virus, can be distinguished from those which are excited by other causes.

Mr. Lawrence endeavours to account for the remarkable fact, that two surgeons of such talent and industry, both of whom had been long carefully investigating the venereal disease, were unacquainted with morbid affections of so serious a nature, and such strongly marked characters, by supposing that in their day all diseases of the eye were given up to a separate class of practitioners. We are not altogether contented with this explanation, although we confess we are not prepared to offer another more satisfactory. We cannot bring ourselves to believe that two such zealous and patient inquirers into all the phenomena of disease, men who were so eager to improve their art, could have indolently given up to the care of others cases of the kind referred to, if they ever fell under their observation. On the other hand, we know no reason why venereal affections of the eye should have been less frequent then than in the present day. There appears to us to be some mystery upon this point, which requires still further elucidation. We now proceed with our task of analysis.

*Acute gonorrhœal inflammation of the Conjunctiva* is the first subject of consideration. Three distinct forms of ophthalmic inflammation occur in conjunction with, or dependence on, gonorrhœa: acute, and mild inflammation of the conjunctiva, and inflammation of the sclerotic coat, sometimes extending to the iris. The first species is a violent inflammation of the mucous membrane of the eyeball and lids, attended with a profuse discharge of fluid, apparently resembling that which issues from the inflamed urethra in gonorrhœa, "and occurring in some kind of connexion with that complaint." It is the most violent and rapidly destructive inflammation to which the eye is subject, and fortunately it is one of the most rare. The organ is often irreparably injured before the patient seeks for surgical relief.

"Purulent ophthalmia, in its various forms, commences in, and is at first confined to, the mucous membrane of the eye. It soon extends to the cornea, which it either disorganizes or changes in structure so considerably, as frequently to destroy or seriously injure sight. The whole texture of the inflamed conjunctiva swells; its blood-vessels are distended to the highest degree, and the membrane becomes of an intense bright red. The texture of the mucous surface is loosened and softened; it becomes pulpy and granular; in short, very much like the secreting surface of some parts of the alimentary canal; and, in this altered state, it pours forth the puriform discharge, which is not produced by suppuration, but is merely the ordinary mucous exhalation, altered in its qualities, and increased in quantity by the inflammation of the membrane. Indeed, purulent ophthalmia does not in general preduce suppuration: the changes which it more commonly causes in the cornea, are sloughing, ulceration, and interstitial deposition ending in opacity." (P. 11.)

Acute gonorrhœal ophthalmia presents all the characters of purulent inflammation of the conjunctiva in their fullest developement: there is the greatest degree of vascular congestion, intense and general external redness, excessive tumefaction of the conjunctiva, great chemosis, corresponding swelling of the palpebræ, and profuse yellow discharge. The first stage of the disease is short: the inflammation is then confined to the conjunctiva, and is attended with soreness and stiffness, a sensation of sand or dirt in the eye, and some uneasiness on using the organ or exposing it to light. The affection soon extends to the cornea, with agonizing pain in the globe, orbit, and head, augmented to intolerable suffering on exposure to light, and with symptoms of inflammatory fever. The danger to the organ is now imminent, and, when the disease has thus advanced from the mucous membrane to the globe itself, its destructive consequences can hardly be entirely averted by any kind of treatment. The great chemosis and swelling of the lids make it often difficult, or even impossible, to get a clear sight of the cornea. As a guide to our prognosis, it is desirable to do this when we first see the case; "but we should not persist in our efforts at the risk of augmenting pain and inflammation." Although the pain is generally most severe when the dense and unyielding texture of the cornea is the seat of inflammation, cases occasionally occur, of which Mr. Lawrence details examples, in which little or no pain is experienced. The immediate effects of the inflammation on the cornea are sloughing, suppuration, ulceration, and interstitial deposition. The more remote consequences are, escape of the humours and collapse of the globe, obliteration of the anterior chamber and flattening of the front of the eye, staphyloma, prolapsus iridis, obliteration of the pupil, corneal opacity, and anterior adhesion of the iris.

"The eye is sometimes said to burst; a paroxysm of excruciating pain is suddenly terminated by a sensation of something giving way; a little fluid runs down the cheek, and great relief is experienced. It is found that, in consequence of the changes of the cornea just mentioned, the anterior chamber is penetrated." (P. 17.)

After describing the various appearances presented in the cornea before it sloughs, Mr. Lawrence observes that

"Suppuration of the cornea may be general or partial: it is usually the former. The cornea first becomes white, and then assumes a yellow colour. The effused substance is not a fluid, nor is it collected into a cavity; it is a thick viscid matter deposited in the texture of the cornea. Ulceration takes place, and exposes an opaque yellow substance, which looks like ordinary matter, but it cannot be wiped off. The ulcerative process extends until this is removed. If the whole cornea should be destroyed, the humors may escape, and the globe will shrink. Or, the humors may remain, and the tumid conjunctiva scleroticæ contract from the circumference towards the centre of the space left vacant by the cornea, until it completely fills that space, when the eye appears like a red fleshy mass, in which even the original situation of the cornea cannot be distinguished. The ulceration of the suppurated cornea may penetrate the anterior chamber at different parts, at each of which the iris may protrude, the front of the organ remaining ultimately flattened.

"When ulceration takes place without previous suppuration, it generally attacks the margin of the cornea, and extends rapidly through the laminæ, so as to form a deep trench, seldom occupying less than one third, often one half or two thirds of the circumference, and sometimes extending round the whole circle. In the latter case, the portion insulated by the ulcerative process sloughs. On the sides of this ulcerated trench, the laminæ of the cornea may be often seen very distinctly. Beer says that they turn up like the leaves of a book which has been much read. If the ulceration should not occupy more than two thirds of the margin, the vascular supply of the cornea will still be carried on, and the mischief may be repaired. As the margin of the cornea is covered by the swollen conjunctiva, these ulcers are at first concealed from view, and we do not know of their existence until the chemosis begins to subside. When the ulcer has gone through the corneal laminæ, the membrane of the aqueous humor may rise as a transparent vesicle in the cavity; or it may be pushed forwards by a protruding portion of the iris. The ulcerative process may penetrate the anterior chamber, when the iris will either fall against the opening, or be pushed into it and block it up. If the ulcer, whether it should have arisen from the separation of a slough, or have occurred in the manner just described, should be spreading, the inflammation remaining unchecked, its surface is whitish, and ragged, or flocculent; or of a dirty yellowish cast, with surrounding haziness. When the inflammation subsides, it becomes transparent. The commencement of the restorative process is marked by the surface of the excavation assuming a light greyish tint, with a jelly-like appearance. A soft semi-opaque substance slowly fills up the breach, when the surface becomes smooth, and the regular figure of the cornea is restored. No secretion of pus is observed, either during the stage of ulceration or that of reparation; the latter process is slow, several days often elapsing without any sensible change in the size or appearance of the ulcer. The same process of contraction takes place here as after the cicatrization of other ulcers, so that the size of the opaque cicatrix is much less than that of the previous corneal ulcer; and as these ulcerations take place on the circumference of the part, one that has been of considerable size leaves a mark that is only observable on close inspection, while, where the ulceration has extended over the edge of the pupil, the cicatrix may leave that aperture quite unobstructed.

"The existence of the ulcer makes no difference in the kind or degree of pain during the active period of the disease: no pain is felt when the inflammation is stopped, although a large ulcer may still exist." (P. 20.)

Various cases are detailed in the work, illustrative of the different changes in the cornea, which are here described. When interstitial deposition takes place, the corneal laminæ are the seat of the mischief; and the opacity thus produced is of the dense kind, called *leucoma* or *albugo*. It is usually accompanied with anterior adhesion of the iris, *(synechia anterior.)* Contraction or obliteration of the pupil may occur in consequence of protrusion of the iris in partial staphyloma, or at the smaller apertures produced by ulceration, or of its adhesion to a leucomatous portion of the cornea. Total staphyloma sometimes results.

*Diagnosis.* The local symptoms establish no distinction between this affection and violent common purulent inflammation. Its peculiar nature is indicated by the preceding or existing gonorrhœa. In general it attacks only one eye, while purulent ophthalmia affects both. Dr. Vetch,* speaking of the latter, says, "there is not one case in a thousand in which one eye only becomes affected." The symptoms of purulent ophthalmia are not so severe, rapid, or destructive.

*Prognosis.* The sight of the patient is always in danger. If we see the complaint in the first or second stage, we may expect to arrest its progress by active treatment, but success, even under such favorable circumstances, is not certain. If the cornea possess its natural clearness, the eye may be saved. When it becomes hazy and dull, or assumes a white nebulous appearance, consequences more or less serious will inevitably ensue. The higher the inflammation, the greater the danger. The changes to which the cornea is liable do not always destroy sight; their effect depends on their extent. Sight may be restored after partial sloughing of the cornea, and extensive ulceration may occur without injury to vision. Mr. Lawrence relates cases of this kind. When both eyes are attacked in succession, the disease is less severe in the second, which therefore is usually saved.

"*Causes.* In investigating this part of the subject, we have to inquire what is the nature of the connexion between this inflam-

* On Diseases of the Eye, p. 195.

mation of the eye and gonorrhœa; whether the former can be produced by the application of gonorrhœal matter to the organ? If so, whether an individual can infect himself? whether the application of matter from another source be necessary; or whether the infection may occur in both ways? Whether, on the other hand, gonorrhœal ophthalmia may be an example of that peculiar transference of diseased action which is called metastasis? To some it may appear necessary to examine a previous question; viz. whether there is any connexion at all between the inflammation of the urethra and that of the eye?" (P. 27.)

To the negative testimony of Mr. Pearson upon the latter point, which we have already mentioned, Mr. Lawrence opposes the positive experience of many competent observers, and the facts he details himself. Beer* and Scarpa† incidentally state, that if gonorrhœal matter be applied to the eye, it excites only a slight degree of inflammation: but we are not furnished with the proof on which this assertion rests. Dr. Vetch took matter from the eyes of persons labouring under acute purulent ophthalmia, and applied it in each case to the urethra of the same individual. No disease was excited: but, when he applied the same matter to the urethra of a different individual, it produced a very virulent gonorrhœa.

"He infers from these experiments, that gonorrhœal matter taken from the urethra and applied to the eye of the same individual would excite no inflammation of the eye. The inference is probable, but not necessary. Because the purulent secretion of the eye does not affect the urethra, we cannot conclude that the gonorrhœal secretion of the urethra will not affect the eye. These morbific influences are not in all instances reciprocal: inflammation of the urethra often causes inflammation of the testicle, but the latter seldom or never produces the first. Dr. Vetch further mentions, that an hospital assistant, named Smith, applied gonorrhœal matter to his own eyes with impunity. When we consider how this matter is diffused over the linen of patients, both male and female, how often the fingers must be smeared with it, and how inattentive to cleanliness the lower classes are, we cannot help concluding that gonorrhœal discharge must be often applied to the eyes of the same individual; yet gonorrhœal ophthalmia is comparatively rare." (P. 30.)

But, although these various considerations would lead us to expect that gonorrhœal discharge would not affect the eyes of the same individual, cases are met with in practice from which there is every reason to draw a contrary conclu-

* Lehre, v. 1, § 540.

† Treatise on the principal Diseases of the Eye. Translated by Mr. Briggs. Second Edition, p. 164.

sion. Mr. Lawrence details examples he himself witnessed, and mentions others that have been communicated to him by his friends, in which acute gonorrhœal ophthalmia clearly arose from the application of the infectious matter to the eyes of the same individual. Gonorrhœal ophthalmia may certainly be produced by the application of gonorrhœal matter from another person. For obvious reasons, this cannot be a very frequent occurrence, and Mr. Lawrence has seen no instance of the kind. But he says,

"Mr. Wardrop has furnished me with two examples. An old lady went into the dressing-room of her son, who had gonorrhœa, and washed her face with a towel which he had been recently making use of. Purulent ophthalmia quickly supervened, and destroyed the eye in a few days. A washerwoman, who had been employed in cleansing foul linen, was seized in a few hours with puriform ophthalmia, which terminated in the suppuration and collapse of both eyeballs. Delpech* mentions the instance of a young and healthy woman, who washed her eyes with goulard water and a sponge, which had been used by a young man affected with gonorrhœa. Violent ophthalmia came on, and quickly terminated in loss of the eye. Mr. Bacot† distinctly traced the origin of the disease to infection by means of matter from another individual, in three instances, two of which were washerwomen." (P. 32.)

But, in a great proportion of these gonorrhœal ophthalmia, we cannot trace the disease of the eye to the application of infectious matter, either from the same or another individual. The eyes are said to suffer by metastasis: it is stated that the gonorrhœal discharge is suppressed, and that the inflammation of the eyes occurs in consequence. Such is the opinion of Richter,‡ Scarpa,§ and Beer,‖ who consider the restoration of the discharge from the urethra a principal indication in the treatment of the disease.

"In none of the cases, which have come under my own observation, has the urethral discharge been stopped: although it has generally been lessened, it has continued in some with little diminution.** On the other hand, the sudden stoppage of gonorrhœa, when effected by surgical treatment, is not followed by inflammation of the eyes. Since, then, gonorrhœal ophthalmia may occur while the discharge from the urethra continues, and since it does not take place when that discharge is stopped, we cannot admit that the affection of the eye owes its origin to the cessa-

* Chirurgie Clinique, t. i. p. 318.

† Treatise on Syphilis, p. 132.

‡ Anfangs-gründe, v. iii. § 57.

§ Treatise, &c. p. 162-3.

‖ Lehre, v. 1, § 533.

** Delpech gives nearly a similar statement. "Il est bien reconnu que l'écoulement ne cesse pas toujours en pareil cas; que quelquefois, et même assez souvent, il subsiste dans toute sa force.—*Chirurgie Clinique*, v. i. p. 319.

tion of disease in the urethra. I am inclined to refer its occurrence to the state of the constitution, without being able to point out in what that state consists; and to regard it as a pathological phenomenon analogous to those successive attacks of different parts which are observed in gout and rheumatism. The two other forms of ophthalmic inflammation which take place in conjunction with gonorrhœa, show themselves only in rheumatic subjects, and generally in connexion with other arthritic sufferings; and the difference between one of these and the affection now under consideration is only in degree. This view of the subject may throw some light on the circumstance that, though direct infection operates equally on both sexes, the gonorrhœal ophthalmia, said to originate in metastasis, seems to be confined to the male. I have never seen it in the female; and Beer, in the passage last quoted, says that he has observed it only in young, robust, and plethoric men.

"The state of constitution, whether hereditary or acquired, which leads to gout and similar affections, is much less common in women than in men; and will hardly be found at all among those young and previously healthy females who are the principal subjects of gonorrhœa. Again, the morbid influences which are experienced and exerted by the male urethra, are different from those of the vagina." (P. 34.)

*Treatment.* Mr. Lawrence is of opinion that the boldest and most prompt antiphlogistic treatment is necessary. Blood, he says, must be drawn freely and at short intervals, both generally and locally, until vascular congestion is relieved, and pain removed. He thinks that as much blood should be taken from the arm as will flow from the vein, and that the evacuation should be repeated as soon as the state of the circulation will allow us to get more.

"These (says Mr. Bacot,) are cases which defy all the usual etiquette of regular and ceremonious visits. If we wish to save our patient from the destruction of his vision, we must scarcely depart from his bedside until the inflammatory symptoms are controlled. The lancet must be hardly ever out of our reach; for if ever there was a disease in which blood may be taken away without limitation, it is this." (P. 36.)

Cases given in the work are referred to, to prove the extent to which the abstraction of blood is often necessary. Local bleeding will suffice for the slighter symptoms which may show themselves after the inflammation has been subdued. The more vigorous depletion is required where the inflammation is fully developed, without the cornea being affected; or where the condition of the cornea may be doubtful; where, in fact, we may hope to save the organ from all injurious change.

"If sloughing or suppuration should have already occurred, it will be of no use to pursue this very active treatment, although more moderate depletion may still be necessary. General sloughing, or general suppuration of the cornea, is usually attended by diminution of the inflammation and cessation of pain, or at least comparative ease: the loss of blood therefore is no longer required for the relief of suffering; and it would be without an object, as vision is irreparably destroyed.

"But inflammation may continue with undiminished violence after the occurrence of partial sloughing; and active depletion may still be necessary, both to limit the extent of the mischief and to favor the processes of separation and restoration. In Cases 8 and 9, very free depletion, both general and local, was employed after the cornea had suffered partially in this way; and the treatment was completely successful in preserving sight. In Case 3, where one cornea had sloughed entirely, and the other eye was actively inflamed, the venesection and local bleeding employed on account of the latter had no prejudicial effect on the former." (P. 38.)

Mr. Lawrence does not place much reliance upon blisters. The ordinary local applications may lessen suffering, but will not check this violent disorder. Some patients find the warm most beneficial, others cold, and the feelings of the patient must be our guide. The eyelids and cheek must be frequently cleaned. When the inflammatory symptoms have been quickly and completely subdued, the effects of the disturbance will pass off in a little time, without the use of astringents and tonics. It will be sufficient to lessen the restrictions in diet, and to use mild aperients. Sloughing of the cornea sometimes, but very rarely, is attended with evidences of general depression, requiring tonics and cordials.

"Such symptoms may occur in conjunction with an unfavorable state of the corneal ulceration, in which, although the swelling and redness of the conjunctiva are diminished, the ulcer spreads with a whitish or dull yellow colour, and an irregular surface and edge." (P. 41.)

Here bark and more generous diet are necessary, and astringent lotions may be proper. They must be used with caution, however, we are told, for fear they should cause a relapse of the inflammation, which must have been subdued before they can be safely employed. The best forms of astringent applications are, the solution of alum, from two to ten grains to the ounce of water; solution of nitrate of silver; or undiluted Liq. Plumb. Sub. The use of a powerful astringent has been recommended in the very commencement of the affection, as a means of cutting it short, and preventing the developement of the inflammation.

Very strong testimony in favor of the astringent plan of treatment in ordinary purulent ophthalmia is given by Mr. MELIN,* Drs. RIDGWAY, O'HALLORAN,† &c. In several very acute cases of this kind, we have been astonished at the rapidly beneficial effects of the nitrate of silver ointment, which was directed by Mr. GUTHRIE at the Royal Westminster Infirmary for Diseases of the Eye; and we are persuaded that Mr. Lawrence's apprehension of increasing inflammation by such treatment is quite unfounded. But let it be remembered that much depends upon the ointment being carefully prepared, and applied according to Mr. Guthrie's directions.‡ In the instances which we have seen, blood was always abstracted, but in moderate quantities.§

Mr. Lawrence has employed the nitrate of silver (in solution) in two cases of conjunctival inflammation, with the best result. One of these was mild gonorrhœal inflammation; the other was catarrhal inflammation of the membrane affecting both eyes of a gentleman, who had been convalescent from gonorrhœa for a few weeks. In the latter instance it must be observed that *three pounds* of blood were taken from the arm, and free purges given before the local application was employed.

As destructive or injurious consequences have so frequently resulted under the usual management of this disease by very free bleeding, &c. Mr. L. would "certainly employ the local astringent, if he met with a case favorable for the trial: that is, where the affection had not extended beyond the conjunctiva. Bloodletting might be resorted to at the same time. In most cases, however, our aid is not sought until the cornea has become affected, and it is therefore too late for the astringent plan." From what we have seen of this mode of treatment, we are convinced it is not necessary thus to restrict its employment. In the various reports we have published of cases at the Royal Westminster Ophthalmic Hospital, several examples will be found, in which, although the cornea was highly inflamed and ulcerated, the Arg. Nitr. ointment quickly relieved the symptoms, but moderate quantities of blood being abstracted.|| The case of Catherine Bulkley** is an excellent illustration of the advantages of this practice, which Mr.

* London Med. and Phys. Journal, vol. lii. p. 184.

† Practical Remarks on Acute and Chronic Ophthalmia, &c. 1824. Part i. ch. i.

‡ London Med. and Phys. Journal, September 1828, p. 193.

§ Ibid. December 1829, p. 521.

|| A case in point is given in our Hospital Reports of this Number, p. 315.

** London Med. and Phys. Journal, December 1829, p. 521.

Guthrie so frequently employs. Here there was great chemosis of the ball, encroaching upon the edges of the cornea, together with superficial ulceration and some degree of loss of transparency of that part; considerable purulent discharge; great pain in the head and eye; discharge scalding hot. The nitrate of silver ointment was immediately applied by Mr. Guthrie himself. Eight ounces of blood only were taken by cupping the first day; no medicine was given: the symptoms speedily subsided. We saw this case from the commencement to its termination, and we thought, when the patient first presented herself, that it was almost impossible her eyes could be saved. This woman had had gonorrhœa for more than a month.

We are surprised that Mr. Lawrence has not adverted to the extensive experience of Mr. Guthrie upon this subject, as he has contributed very much to establish the efficacy of the astringent plan of treatment in acute and chronic ophthalmia.

The influence of mercury in gonorrhœal ophthalmia is doubtful. Mr. Lawrence has seen both the ordinary purulent and gonorrhœal ophthalmia proceeding, apparently unchecked, under a full mercurial action. Beer* asserts that mercury is of no service; and Delpech speaks to the same effect.† On the supposition that gonorrhœal inflammation of the conjunctiva depends immediately on the suppression of the discharge from the urethra, the restoration of that discharge has been made a main point in the treatment by some surgeons of experience and judgment, as Richter,‡ Scarpa,§ and Beer.||

"In spite of the confidence which one is inclined to repose in the practical knowledge and judgment of those whose advice has just been quoted, I cannot help thinking that the measures in question have been recommended rather on theoretical grounds than from experience. At least, these writers do not mention any results of their own practice; nor have I met with any cases in which the employment of such means is mentioned. In none of the instances which have come under my own observation has the gonorrhœal discharge been suppressed, so that the reason for this kind of practice has not existed. Again, when the violence and rapidity of the disease are considered, in contrast with the slow-

* Lehre, edit. 2, v. 1, § 539.

† Chirurgie Clin. de Montpellier, v. 1, p. 321.

‡ Anfangs-gründe, v. 3, § 58-61.

§ Treatise, &c. 2d Edition, p. 166.

|| Lehre, v. 1, § 537-539.

ness and uncertain operation of this treatment, we cannot doubt that irreparable mischief would be done to the organ during the time lost in such attempts." (P. 49.)

*Mild gonorrhœal inflammation of the conjunctiva* "may be safely and advantageously treated on the astringent plan, particularly by the solution of lunar caustic, the use of which may be preceded by antiphlogistic means in patients of full habit, or where we fear that the organ is in danger from probable extension of inflammation to the cornea.

*Gonorrhœal inflammation of the external Tunics and Iris.* The vascular trunks lying between the conjunctiva and sclerotica are distended, and the anterior portion of the latter membrane becomes of a pink or purplish red. As the conjunctiva participates but slightly in the affection, these changes are distinctly seen through it. There is increased lachrymal secretion, severe pain in the eye, with sense of tension, intolerance of light, with profuse discharge of tears on the slightest exposure.

"The inflammation soon extends to the iris, which loses its brilliancy, assuming a dull and deeper hue. The pupil contracts, and lymph is effused from its margin. The external redness is increased, the vessels of the conjunctiva being more distended. The cornea at the same time becomes hazy, and vision is more or less impaired. Nebulous opacity and speck of the cornea are sometimes produced. See Cases 15 and 24. As the inflammation subsides, the iris recovers its natural colour, and vision is restored.

"If the inflammation be considerable, it may cause adhesions of the pupil, with contraction of the aperture; and the adhesions thus formed are sometimes white, as in arthritic iritis. Even permanent dimness of sight may be produced. Sometimes repeated attacks of the disease occur, each of which causes fresh adhesion, so that at last the pupils are fixed in their whole circumference, and considerably contracted. This is exemplified in Case 16, which also shows that the complaint is not always very serious, as the patient had escaped without any material imperfection of sight, although he had employed nothing but a wash in the several inflammations he had experienced.

"This affection must be treated by the abstraction of blood, general or local, and by other corresponding measures. If the inflammation be considerable, if it should occupy both eyes, and the patient should be young, robust, and plethoric, free general bleeding will be required. Cupping and leeches will suffice in the milder instances. Warm local applications are generally the most agreeable to the patient's feelings: the poppy fomentation answers the purpose very well. Exclusion of light is absolutely necessary so long as the intolerance continues. When the inflammation is checked by these measures, blisters may be advantageously applied, and the cure may be completed by the administration of

Plummer's pill once or twice a day, with mild aperients and a regulated diet. Colchicum is often used with advantage on account of the rheumatic symptoms which accompany this affection; and the eye may be expected to participate in the benefit, although the remedy cannot be depended on as a means of counteracting dangerous inflammation of the organ. The same observation is applicable to residence at the seaside and warm bathing, which are more advantageous to the lingering arthritic ailments under which patients frequently suffer so long in these cases, than to the ophthalmic affection." (P. 53.)

Patients often experience at different times, "in consequence of, or in connexion with gonorrhœa," the above kind of inflammation and mild inflammation of the conjunctiva. Rheumatic inflammation of the joints accompanies both these forms of ophthalmic disease. Mr. Lawrence relates cases of this kind, and refers to others which have been published by Mr. Brodie* and Dr. Vetch.† Inflammation sometimes exists at the same time in the urethra, the eyes, and joints: in other instances these parts are affected successively. Several examples of this form of disease are given.

"The affection of the eye last described is exactly the same as rheumatic inflammation of the sclerotica and iris occurring independently of gonorrhœa. Both this and the mild purulent inflammation of the conjunctiva are to be regarded as rheumatic affections of the organ excited by gonorrhœa: that is, they take place in individuals, in whom this constitutional disposition is shown by inflammation affecting either the synovial membranes or the fibrous structures of several joints. Although the organs seem at first view very dissimilar, there is an analogy of structure between the parts which suffer in the two instances; that is, between the synovial membranes and the conjunctiva, and between the ligaments and fibrous sheaths and the sclerotica. Hence we need not be surprised at finding that the eyes suffer under the influence of that unsound state of constitution which leads to these affections of the joints. The structure originally affected, the lining of the urethra, is also a mucous membrane, which sometimes becomes inflamed, and pours out a puriform discharge, in gouty and rheumatic subjects, from internal causes. That the essential cause of this combination of morbid phenomena is peculiarity of constitution, may be inferred from the repetition of attacks, and the length of time for which some individuals are harassed by successive appearances of disease in various parts." (P. 58.)

In the nineteenth case detailed by Mr. Lawrence, discharge from the urethra *without* infection occurred four times; then inflammation of the foot; three years after,

* On Diseases of the Joints, pp. 55 and 60.

† Practical Disease, &c. p. 243.

severe inflammation of the chambers of the aqueous humor; then gonorrhœa and mild purulent inflammation of the conjunctiva, followed by rheumatic inflammation of various joints; and afterwards severe rheumatic inflammation of the sclerotica and iris. One patient seen by Mr. Brodie had undergone four attacks, all of which began with gonorrhœa: it was followed first by purulent ophthalmia, and then by inflammation of the synovial membranes of several joints.* This train of diseases must be referred principally to peculiarity of constitution; gonorrhœal infection is not essential to their production: it is only to be regarded as one of the exciting causes, and perhaps the most frequent.

Mr. Lawrence details twenty-four cases of gonorrhœal ophthalmia; fourteen of them were of an acute form. "Loss of vision took place in *nine* from sloughing, suppuration, or opacity of the cornea. In two of these an eye was lost, and the other recovered. Sight was restored in the other five, with partial opacity of the cornea and anterior adhesion of the iris in three of the number." In some of these instances the eye had sustained irreparable injury before Mr. L. was consulted. But still quite sufficient evidence remains to show that even the prompt and active application of the antiphlogistic plan of treatment is followed by very discouraging results. We have seen several cases, and we know of others, in which from a very early period of the disease, this practice was adopted to its fullest extent, compatible with the safety of the patient, and still destruction of the eye ensued.

Gonorrhœal ophthalmia differs only from common acute purulent ophthalmia in the greater degree of its severity, and the rapidity of its destructive progress. In the latter disease, abundant experience has proved the superior efficacy of moderate bleeding, conjoined with the astringent plan. At the Westminster Infirmary for Diseases of the Eye, the practice of copious and repeated bleeding has been carried to its utmost extent, and abandoned from the conviction of its utter incapability of arresting the disease. Mr. Guthrie now pursues a different practice. From ten to twenty ounces of blood are abstracted, and the strong nitrate of silver ointment is immediately applied, even during the acute stage of the disease, with much greater success.† We have seen several very acute cases at

* On Diseases of the Joints, p. 63.

† Mr. Bacot (Treatise on Syphilis, p. 138,) relates an interesting case in which gonorrhœal ophthalmia was very speedily cured by the application to the eye of a solution of lunar caustic, ten grains to an ounce.—Ed.

this institution treated in this manner, and the severity of the symptoms have been speedily checked. We think we are justified in concluding that the practice which is most advantageous in acute purulent ophthalmia, will be so in the gonorrhœal form of the disease. Mr. Lawrence, we lament to say, is not yet discouraged even by the frequent failure of bold antiphlogistic treatment. He infers, not that this practice is incapable of arresting the inflammation, but that it has not been employed to a sufficient extent. In support of this opinion he refers particularly to his fifth case. In this instance the patient was seen "in the earliest period of the affection;" "the most active treatment was adopted, in spite of which, however, the eye was lost." Now, as the *most* active treatment was here promptly applied, we are surprised that Mr. Lawrence should regret it had not been carried to a still greater extent. We should have expected that the unfortunate result of this case alone would have induced him afterwards to try the other practice, so confidently recommended by many surgeons of extensive experience. We most earnestly recommend him to give the astringent plan of treatment a fair trial. The evidence in its favor is too strong to be rejected; and, even if extended experience should show that it is only equally successful with the practice of very large and repeated bleedings, it ought to be preferred. The constitution of the most robust patients not unfrequently suffers permanent injury from such severe practice; and their recovery is in most cases very tedious.*

In the next Number we shall notice the second part of Mr. Lawrence's work on "Syphilitic Diseases of the Eye."

---

*A Treatise on Deformities; exhibiting a concise View of the Nature and Treatment of the principal Distortions and Contractions of the Limbs, Joints, and Spine. Illustrated with Plates and Woodcuts.* By LIONEL J. BEALE, Surgeon. —8vo. pp. 248. Wilson, London, 1830.

THE subject to which Mr. BEALE directs our attention, although of the utmost importance both as regards the health and happiness of numerous individuals, has been

* We have often heard the astringent, or, more correctly speaking, the stimulant plan of treatment objected to in acute ophthalmia, upon hypothetical grounds, by those who confessedly had never put its efficacy to the test in practice. The eye, it is said, is highly inflamed, and therefore stimulating applications must be injurious. It would be quite sufficient to reply, that experience has proved the contrary : but we may go a step further, and defend the practice upon principle ; for the success of the stimulating treatment, together with *moderate* bleeding, in acute inflammation of the eye, very clearly illustrates the solidity of those pathological doctrines of inflammation which have been so ably and ingeniously maintained by Drs. Wilson Philip, Hastings, and others.—ED.

comparatively neglected by regular surgeons. The treatment of deformities, until recently, has usually been confined to the empiric or mere mechanic. From neither could much benefit be expected, but it is probable that the latter has inflicted the most positive injury. In the treatment of deformities, the surgeon may undoubtedly obtain much assistance from an expert mechanic: he may construct his instruments ingeniously, but he cannot adapt them to each particular case. The scientific surgeon alone can determine the cause of the deformity, or the indications to be fulfilled in attempting to remedy it. The frequency of various kinds of distortion, and the obvious necessity for improvement in their mode of management, have at length attracted the attention of the profession, and if the public are any longer imposed upon by unprincipled adventurers, they have themselves to blame. Many valuable monographs, and scattered essays in different medical periodicals, have been published upon the subject of deformities; but still we have no work which professes to treat generally of such maladies. The task which Mr. Beale has undertaken is not therefore unnecessary: his object being to combine the experience of former writers, with the facts he has himself observed, relating to the nature and treatment of the principal deformities to which the human frame is liable.

It is stated in the preface that much unfounded prejudice prevails among English surgeons, with regard to the employment of mechanical apparatus in the treatment of distortions of the limbs. We believe that it is only the *injudicious* employment of such means that has been objected to, and that Mr. Beale will find few, if any, of his surgical brethren who are not inclined to admit with him that many distortions cannot be removed without the aid of mechanical instruments. As a proof of the efficiency of such instruments, Mr. Bransby Cooper's account of the osteology of a Chinese woman's foot is referred to, and it is said that "if such deviations may be effected contrary to nature, we may confidently apply mechanism to place parts in those situations which nature intended they should occupy." We do not conceive that this is a happy illustration of the probable advantages of mechanical means in cases of deformity. It by no means necessarily follows, that because the natural growth of a limb may be checked and distorted by artificial manœuvres, a deformed member, of which most likely all the parts are more or less enfeebled or diseased, should by the same means be restored to its natural position. The point is trifling, perhaps, and we only notice it because we think that Mr. Beale has selected a very weak argument in recommendation of the practice he is very properly anxious to defend.

In an introductory chapter a neat anatomical sketch is given of the various parts which are implicated in distortions. In his general observations, the author justly observes that there is no notion more pernicious than the supposition that children will outgrow deformities. A deformity may stop at a certain point, and, as the body grows, become less apparent; but the instances of a natural cure are so few, that all such hopes ought to be abandoned, and proper means of cure resorted to at as early a period as possible.

"Children with distorted limbs are sent from their homes to the sea, in the hope that, by increasing the vigour of their constitution, the deformity may be removed; but disappointment too generally follows, and the malady, augmented during the time so devoted, must sooner or later be met by the only certain methods of cure,—mechanical assistance, and means which give tone to the faulty muscles." (P. 39.)

With regard to the period of life to which a possibility of curing deformity extends, there will of course be great difference according to the cases. The earlier the stage in which curative means are applied, the better; but it may be established as a general rule, that, during the period of growth, most distortions, that are at all curable, may be entirely removed.

"So long is it before any marked effect is produced on the form of the bones, in many varieties of club-feet, that these distortions may be entirely effaced, at almost any period before growth ceases. Even after the period of youth, they may be much benefited, and sometimes altogether removed. The same observations apply to many varieties of distorted spine. To be able to give a fair prognostic, we must be very careful in distinguishing the true nature of the cause, and the degree to which it has operated on the limbs or spine. In inflammation of the fibro-cartilages, for example, if the disease has made such progress as to cause atrophy, or absorption of the cartilages, or if they are covered with ossifications, we can never hope to restore the form, which, in the earlier stages of this variety, may with certainty be predicted. It is of much importance to ascertain whether the deformity is still making progress, because, if we find that for the space of a year or two there has been no advance, but a complete and gradual cessation of the attendant symptoms, it will be a strong presumption that the time for a perfect cure has gone by. This opinion will be confirmed, if we find that extension produces no effect." (P. 40.)

In applying extension to the spine, the utmost circumspection is necessary. The accidents which may be produced demand that such means should never be employed but with the greatest prudence, and after a most careful investigation has clearly distinguished the species of the malady. In the chapter on Rickets, Mr. Beale remarks that "the upper extremities are always less deformed than the lower; the bones are bent from muscular action *alone,* and acquire a direction forwards from the preponderating influence of the flexors." We doubt the correctness of this statement. The bones are not bent by muscular power alone: the subjects of rickets, on the contrary, are precisely those who are most deficient in muscular vigor. The primary cause appears to be non-assimilation of food, occasioning imperfect nutrition; diminished healthy circulation; a consequent weakness in the muscles; and defective solidity of bone. The lower limbs will be more especially liable to bend, as they have to bear the weight of the trunk. The opinion that rickets is attributable to defective solidity of bone, is corroborated by a comparison between the specific gravity of healthy bone, and that of the bones of those subject to this malady; the former being much heavier than the latter. Mr. Beale himself, indeed, states, in the first sentence of the chapter, that in rickets the bones lose their tenacity and hardness, and bend under the weight of the body, and are also distorted by the action of muscles.

"*Diagnosis.* There is no disease which can be confounded with rickets; but it is important to distinguish curvatures of the spine, produced by this malady, from those originating in other causes. The application of mechanism, occasionally useful in some cases of spinal distortion, would obviously be improper where the bones are preternaturally soft; the pressure of instruments on the pelvis would tend further to increase any deformity, which might have already taken place in that part.

"We should carefully examine the effects produced by the malady in other bones, besides those of the vertebral column. Not only do the bones of the extremities become soft and flexible, but those of the thorax and pelvis are more or less diseased. The pelvis in this malady seldom fails to partake the fate of the spine; but in deformed spine, from other causes, the bones of the pelvis are but rarely affected. This is a most important distinction, and by properly attending to it, in obstetric practice, the alarms of many families may be entirely removed. As a general rule, it may be said, that deformities occurring after the first ten years of life, seldom implicate the form of the pelvis." (P. 59.)

The general treatment of rickets recommended by Mr. Beale is such as is usually adopted. Upon one point we hesitate to agree with him. He says that, "during the whole time that the bones remain flexible, the patient should lie in the recumbent position, and daily extension should be applied, to restore or to maintain the proper direction of their course." By keeping a patient constantly in a recumbent position, we should fear to retard rather than accelerate the acquisition of muscular strength, and consequent solidity of bone. Alternate exertion and rest are much more likely to attain the desired result. Exertion must, of course, be in proportion to the strength of the child and the degree of the disease, but absolute inaction can rarely be required, and will be commonly prejudicial.

Deformities of the upper and lower extremities, pigeon or chicken breast, and wry neck, are the subjects discussed in the ensuing chapters. Upon each of these points Mr. Beale gives an abstract of the opinions of the best authorities.

*General observations on Spinal Distortions.* Upon this subject the most discordant opinions have been maintained. By some the original cause has been ascribed, exclusively, to the muscles; by others to the ligaments and cartilages; and, from the time of Pott until very lately, caries of the bones was universally considered as the cause of all the varieties of spinal deformity. Before the time of POTT these diseases were only considered with respect to their external characters, and the distortions were mechanically treated, without any regard to their causes. Even in the present day, mischief is often done by those who, judging alone from external symptoms, profess to cure all deformities by external force. Within the course of the last few years, a rapid improvement has taken place in our knowledge of the pathology and treatment of spinal distortions. Mr. Beale states that we are indebted to Mr. SHAW for the best work on lateral curvature, and for calling our attention to muscular inactivity, and consequent debility, from which this variety of distortion most commonly arises. We fully admit the practical excellence of Mr. Shaw's work; but the merit of first calling the attention of the profession to "muscular inactivity, and consequent debility," in such cases, is due to Mr. WARD, who published a valuable essay* upon this subject, about a year before the appearance of Mr. Shaw's treatise. DELPECH, in France, has carried pathological investigations further than any other writer. His account of curvatures from chronic inflammation, or what he terms a peculiar affection of the fibro-cartilages, and from a tuberculated condition of the ver-

* Practical Observations on Distortions of the Spine, Chest, and Limbs; together with Remarks on Paralytic and other Diseases connected with impaired or defective Motion. By WILLIAM TILLEARD WARD, F.L.S. &c. London, 1822.—Mr. SHAW published his work in 1823.

tebræ, being derived from extensive opportunities of post-mortem inquiries, are particularly valuable.

"By fair analogy, we may expect to find in the joints of the spine the same diseases which attack analogous structures in the other articulations of the body, and any man of unbiassed judgment will readily see that the primary cause of distortions is sometimes seated in the muscles, sometimes in the ligaments or cartilages, and sometimes in the bones. As the mode of treatment is essentially different in these varieties, it is imperative on us to discover the original cause of the mischief: for as it is wiser, and more effectual, to attack the causes rather than the effects of disease, so the trouble of the investigation will amply repay us. At one time it was the fashion to place all persons with deformed spines in the recumbent position, so to remain for one or more years. Now, it is obvious that, if the cause of the deformity lies in a deficiency of muscular power, total inactivity will not be very likely to remedy the disease, and it was therefore no uncommon occurrence to see persons after going through this ordeal, becoming, at the end of the course of treatment, worse than at the beginning; for such a state of disease, the simple remedy is muscular exercise, variously modified. Now the exercises of gymnastics, in cases of inflammation of the fibro-cartilages, or caries of the bones, would clearly increase the cause of the distortion: in these instances, repose in the recumbent position, is the essential feature of the management.

"The earliest source of deformities of the spine is rickets; cases from this cause mostly occur in the first ten years of life. The next general cause is muscular debility, and produces its effect on girls of rapid growth, from the age of ten or twelve to fifteen or sixteen. The affection of the fibro-cartilages attacks children of a lymphatic or scrofulous tendency, during the period of growth; while curvatures from disease of bone occur at all ages, but seldom later than forty.

"Among the occasional causes of deformity, may be mentioned contractions within the chest. The cavity of the chest, like the cavity of the cranium, is entirely filled with its viscera, the parieties are moulded upon their contents, and the proper developement of the thorax depends on the proper developement of the lungs and heart. If any portion of the lungs be deficient, there will be a corresponding defect in the containing cavity. Cases of deformity occur from contractions within the chest, after abscess and destruction of portions of lung. Seurat, the man exposed to the gaze of the curious in this town, under the name of 'the living skeleton,' was an example of this kind. In the work of Delpech will be found a detailed account of this man's case, and many others of a similar kind; Laennec has also described numerous instances of the same nature.

"The contraction of the cavities of abscesses in the lungs draws inward the corresponding ribs, and bends the spine laterally or anteriorly. The tissue generated by suppurative inflammation, which forms the cyst of the abscess, contracts and produces these results. If an abscess forms in the upper part of the lung, the cyst will be attached by adhesive inflammation to the three or four upper ribs, and also to the spine: after evacuation of the pus, the fibrous matter forming the cyst will contract, and bring together the ribs and the vertebræ. This fibrous matter sometimes ossifies, and the deformity becomes fixed and apparent. Recoveries from phthisis and empyema, in this climate at least, are so rare, that we are not likely to have our attention frequently directed to these results.

"Some cases of deformity arise from congenital inequality of the two sides of the body. The muscles, and in some instances the bones, are less developed on one side of the mesial line, than on the other. The most important of these cases is where one lower extremity is shorter than the other: an account of this will be found in its proper place. The undue developement of the bones or muscles of one side of the vertebral column must be so rare a cause of distortion, that we may safely pass it by without further comment.

"Rheumatism is a very common cause of curvature of the spine. In these cases the vertebral column is implicated, and a great arc is formed, the concavity of which is always forwards. The fibrous apparatus on the anterior part of the bodies of the vertebræ is extremely liable to rheumatism; we may also expect, from analogy, that the fibro-cartilages are sometimes the seat of this disease, and the contraction of these parts will necessarily incurvate the spine forwards.

"In subjects predisposed to rheumatism, the slightest twist of the spine will often produce a return of the disease: the writer of of this experienced an attack of lumbago and sciatica, which lasted for months, by suddenly jumping out of bed, and giving the back a jar. In such constitutions a slight deformity of the spine, which has been considered as permanent, will sometimes make a sudden and enormous progress.

"In distortions from rheumatism, the curvature of the spine forwards is, in some measure, the effect of indulgence in the most easy position. Few cases of curvature can be referred exclusively to any one cause, there is generally one or more secondary causes, which operate in conjunction with the primary. So, in rheumatic curvatures, there is generally defective action in the extensor muscles of the back: this operates by allowing an anterior flexion of the vertebral column, on the same principle that it does in the bent back of infancy and of old age." (P. 126.)

The interesting series of papers by Dr. and Mr. D. Griffin which we have recently published, the work of Mr. Teale on Neuralgic Diseases, and other treatises which have lately appeared, show that Mr. Beale is correct in attributing many of the obscure, anomalous, and what are termed nervous cases, which so perplex us in practice, to some morbid condition of the spinal marrow, or the nerves which arise from it. "In such cases it will be wise to refer to that portion of the back where the nerves of the disordered part terminate."

There are three varieties of spinal distortion: lateral curvature, curvature forwards or angular projection, which are degrees of the same inflexion, and curvature backwards. Some confusion has arisen from the use of the same terms, in different senses. The term excurvation, or curvature outwards, has been applied to the common variety of hump back, where the upper part of the spine is bent forwards. Mr. Beale makes use of no other terms than curvature forwards and curvature backwards.

"By the first will be understood all cases where the upper part of the spine is bent anteriorly, by which the sternum is brought nearer to the pelvis than natural: by the latter term will be meant the reverse of this, which, indeed, is so rare a case, that it will require but little consideration.

"In lateral curvatures, the whole spine is more or less distorted: in whatever part the vertebræ may first incline to either side, there will be, above or below, one or more curves in opposite directions, to maintain the equilibrium: and in some instances the head will be so correctly placed over the centre of gravity that the body will appear at first sight to be only shortened, and not distorted. Sometimes the whole of the dorsal vertebræ are included in one great sweep. This variety is generally dependent on muscular debility, and, like all distortions from this cause, is temporary in its incipient stage, disappearing in the recumbent position, and by gentle extension: nor do such cases become truly permanent, until, from very long continuance, the bones or fibro-cartilages are rendered cuneiform by pressure. Curvature forwards is most commonly dependent on disease of the cartilages, or the bones: that degree of it, which has been denominated angular projection, is almost invariably permanent, and depends on progressive, or ulcerative absorption, of the fibro-cartilages or of the bones.

"The spine cannot be deformed in the dorsal region, without implicating the situation, and relations, of the sternum and the ribs: the rotation or contortion which is generally more or less combined with curvature, will also secondarily alter the form of the ribs and sternum. The mode of the posterior articulation of the ribs hardly permits them to escape in any degree of dorsal curvature, unless the ligaments become greatly relaxed: to a certain extent this may occur, but, at a later period of inflexion of the dorsal vertebræ, the ribs corresponding to the concavity of the curve will be necessarily brought nearer together, while those of the convex side will be separated, and on the degree of resistance offered by the ligaments and cartilages will depend the amount of distortion of the chest. The varieties of this are innumerable, as may be seen in any anatomical museum." (P. 132.)

More distortions, perhaps, occur about the two inferior dorsal, and upper lumbar vertebræ, than in any other portion of the spine. The connexion of the vertebræ of the back is firmer than in any other part of the chain: just at the base of the firmer column there will be unusual freedom of motion, and this is the cause of the frequency of distortion in this part. More or less derangement occurs in the functions of the thoracic and abdominal viscera, in cases of spinal deformity. These viscera sometimes, however, accommodate themselves to the pressure inflicted upon them, or to the alteration which occurs in their position, and carry on their respective offices better than might be expected from the degree of distortion which exists. In some cases of spinal deviation, the growth of the body ceases; puberty is retarded; in males, the voice does not change, nor the beard grow.

"When we contemplate the deviations of the viscera of the chest and the abdomen, of the great blood-vessels, of the great centre of the absorbent system, of the various nervous ganglia concerned in the supply of nervous power to the organs of nutrition; when we consider the deranged condition of the spinal cord, the principal seat of sensation and motion, and the centre of communication of all parts with the brain, the perverted course of the nerves connected with it, as well as the great sympathetic; when we call to mind the impaired condition of the respiratory organs, of the intercostal and abdominal muscles; when we remember the important offices of all these parts in the animal economy, we cease to be surprised at the long list of maladies which are attendant on, or consequent to, spinal deformity." (P. 137.)

We may judge of those cases of distorted spine which are susceptible of cure, by gently raising the body by the chin and nape of the neck, or by extension in the horizontal position, which is the best mode in Mr. Beale's opinion. If the curves are in part effaced, it is evident that the vertebræ are not immoveable, and we may therefore give a favorable prognosis. It does not follow that very bad cases are the most difficult of cure; for where there is great but increasing deformity, we have more reason to expect success in our treatment, than when a smaller degree has been long in a fixed state.

"In distortion from caries, the most happy result will he anchylosis of the remaining portions of the bodies of the diseased vertebræ. The necessity of a correct diagnosis in these cases is obvious. The same remark applies to spinal deviations in consequence of rickets; when the disease has terminated, and the bones have recovered their density, any change in form is almost, if not altogether, hopeless." (P. 138.)

The *lateral curvature* is the most common form of spinal distortion, and perhaps there are few delicate females whose figure is not more or less affected by it. It is often so slight as not to attract the notice of the individual. Tight stays, a constant sitting posture, too little exercise, a habit of leaning, or indulgence in any other particular attitudes, may cause a deviation of the spine in the lumbar region.

"In very many instances no further effect is produced, and a very slight curvature in the loins continues for life. It is only when a secondary distortion occurs in the dorsal region, and the figure palpably suffers, that the attention of the patient or her friends is directed to the subject. Perhaps many of the anomalous complaints, to which hysterical and nervous women are so liable, may originate in these slight distortions of the lumbar vertebræ. The affections of the spinal marrow are every day attracting more attention, and I have no doubt that future investigations of this part of the animal economy will lead to the elucidation of these, hitherto, intractable disorders.

"The first symptom which attracts attention, when a lateral curvature is forming, is the greater elevation of one shoulder: this is less perceptible in the morning than in the evening, when the patient is fatigued. By degrees the shoulder rises more, is more full, and a slight lateral bend becomes perceptible in the spine. On examination, the spinous processes of the dorsal vertebræ will be found sweeping too much to the right, those of the lumbar to the left; if a plumb-line be held from the occiput, and the points of the spinous processes be dotted with ink, the degree of deviation will be easily measured. In the dorsal region, the right side of the body will be more full and rounder than the left, which is contracted and depressed: in the lumbar region the reverse condition will be found, fulness on the left, and sinking in on the right." (P. 140.)

When a curvature has been once established, it generally goes on increasing, and a lateral curvature, as it advances, becomes complicated with distortion of the whole trunk.

"In very bad cases of lateral curvature, the form of the pelvis is affected, but this is so rare an occurrence that it must be considered only as an exception to a general rule. The crest of the ileum on the side opposite to the lumbar curvature will sometimes be elevated, the pelvis will consequently be oblique, and some internal irregularity will occur. The younger the subject, the more danger will there be of distortion of the pelvis, which rarely occurs after the age of twelve. When the lower limbs are tortuous, and the spinal curvature is connected with rickets, we may expect to find distortion of the pelvis. The weight and pressure of instruments have been charged with compressing and distorting the pelvis; if applied to the soft bones of very young children, and considerable pressure used, such effects may occur, but in this instance instruments have been accused of more than can be fairly laid to their charge." (P. 146.)

Anatomical investigations have ascertained that the bones are seldom diseased in lateral curvature. When the deformity originates in very early life, they are usually soft and spongy, and lose their shape by pressure; but generally, in this variety of curvature, the bones are never altered in structure, nor is their figure changed but in old and permanent deformities. The muscles and ligaments are much altered, both in form and texture.

The *curvature forwards* is almost as frequent as lateral curvature: it takes place at all ages, but from very different causes.

"The form and the extent of the curvature depends on the number of the vertebræ, or fibro-cartilages, compressed or removed, as well as on the situation of the disease. When the bodies of many vertebræ are destroyed, the curve will approach to an ellipsis or an angle, but the degree of curvature will also depend on the depth to which the bones are corroded. Sometimes many vertebræ are affected, but the osseous matter absorbed or destroyed is not great, because the effects of the disease are confined to the anterior surface of the bodies: hence there may be very extensive absorption, or caries, with little or no deformity. On the other hand, the bodies of one or two vertebræ may be entirely removed, the column will sink at this point, and the spinous processes will be very prominent. We may readily imagine the mischief done in such cases by shampooers and others, who see nothing but dislocation of the vertebræ in such projections." (P. 154.)

The bodies of the vertebræ are seldom entirely destroyed; that portion which forms the anterior part of the spinal canal is the last to be affected, and in most instances escapes altogether, so as to leave the canal continuous and undiminished in caliber. When the distortion has altered the form of the thorax, and reversed the direction of its long diameter, the heart is situated at a greater distance from the spine than natural. Palpitations and irregularity of its action are common, and organic disease of the heart is often suspected.

*Of defective Muscular Action as a cause of Spinal Distortions.* In a state of health, the antagonist muscles of the limbs and trunk are reciprocally poised, and equally proportioned in power. When one or several muscles of similar action lose their power of contractility, the limb is drawn by the antagonist muscles out of its natural direction, and some degree of deformity ensues. In high amputation of the thigh, the flexors place the stump nearly at a right angle with the pelvis, because they have lost their controlling antagonists. As the due maintenance of the osseous connexions is in part dependent on the muscles, a defective or irregular action of these organs must have considerable influence in the production of deformities. The integrity of the entire apparatus of the organs of motion depends upon muscular contractility, and the degree of this is consequent on exercise. There are, however, few cases of spinal distortion which can be exclusively referred to mere weakness of muscle or deficiency of irritability: probably in every case of this kind there is more or less relaxation of the ligaments, and some loss of consistency in the fibro-cartilages; but as these latter states are in some instances consecutive to muscular debility, they may be placed in a secondary rank, as causes of distortion. Muscular debility may arise from various causes, but, in the cases now referred to, it is generally the result of too little exercise; and is therefore a cause as well as a consequence of ill health. The attention which has latterly been directed to this subject has led to the introduction of exercises among young females, which tend to prevent many deformities.

"It is well known that muscles which are not called into action lose their character, and, after some time, suffer almost entire annihilation. It has been asserted than inactive muscles degenerate into cellular tissue, and that the latter is not merely the original tissue in which bone, muscle, &c. are deposited, but even that from which all other parts may be formed. The fact appears to be that, in the process of absorption, the fibrous parts of inactive muscles are removed, and the arteries do not deposit new ones, because they are not required, so that, after some time, little remains in the place of such muscles but fat and cellular tissue." (P. 171.)

Inequality of power in antagonist muscles is a frequent cause of deformity in the limbs and joints. As the limbs on the right side of the body are generally more powerful than those of the left, so this must be in some degree the case with the muscles on the two sides of the spine, and, in persons of weak relaxed habits, may be partly the means of producing a lateral curvature. Deformities occasionally result from paralysis of certain muscles, or sets of muscles. Debility and wasting of some part, often of a whole limb, will be observed; and this may be followed by distortion of spine. Cases of this sort are often consecutive, or coexistent with a deranged state of the nutritive organs, and Mr. Beale believes they readily give way as these organs are restored to health. In spinal distortion from defective or irregular action of the muscles, the attention is first excited by the unusual projection of one of the shoulders or hips, and a disposition to stand or sit on one side. "The pelvis is never deformed in cases originating in defective muscular action." In all distortions of the pelvis, there will be found evidence of rickets, or other disease of the osseous sytem.

"It is well known that ladies, with considerable lateral curvatures of the spine, have very easy labours; and, on the other hand, the pelvis will be very much distorted, where the spine is not at all so. The pelvis will seldom be found affected, unless the distortion of the spine began in early childhood. However twisted or curved the back may be, if the long bones of the extremities evince none of the effects of rickets, we may expect to find the pelvis free from deformity. When the curvature has been established after the age of twelve, the pelvis will seldom suffer, but if the distortion appeared before the age of five, when the effects of rickets usually take place, there will be more probability of finding a narrow pelvis. Mr. Shaw thus expresses himself on this subject: 'I shall even venture to say that, on prosecuting the inquiry further, we shall be induced to conclude, that in whatever state of distortion the spine and ribs may be, the bones of the pelvis will not be found distorted, unless there be at the same time marks of rickets in some of the long or solid bones.'" (P. 181.)

Having described those cases of distorted spine which depend on some defect in the muscular system, Mr. B. proceeds to the treatment.

It will be obvious from the causes enumerated, whether debility, paralysis, or irregular action of muscles, that the indications of relief are in all the cases nearly similar, viz. to give tone and power to the muscular system generally, and to the particularly defective muscles locally. This is to be effected principally by exercise.

"It is of the utmost importance, that a correct diagnosis be formed, because the mode of treatment is essentially different in this, and the two classes of spinal distortions described in the following chapters. Nothing, for example, could be more unwise than to prescribe perfect repose in a class of diseases which are dependent on imperfect muscular action: from carelessness in tracing effects to their causes, and from the system of prescribing for symptoms, this has been done, and I know individuals who have been comdemned to a recumbent position, for months and years, whose cases required to be met with various modifications of exercise. In slight cases of spinal deviation, from mere muscular inactivity, perseverance in a course of exercise, adapted to bring the muscles of the spine into regular action, should be rigidly enforced, not only to restore the vertebræ to their true position, but after this has been accomplished, to maintain them so." (P. 182.)

The exercise employed must be regulated by the strength of the individual, and varied according to each particular case. From well-directed gymnastic exercises of different kinds, the form and vigor of the body, as well as the general health, may be much improved. Mr. Beale admits that the use of machinery to extend the spine has not unfrequently been productive of mischief, from the careless or ignorant manner in which it has been employed; but he contends that "it is a most useful auxiliary, and numerous cases of spinal deformity present themselves, which cannot be benefited without the use of machinery, to overcome the contractions of the muscles, ligaments, and fibro-cartilages." So long as anchylosis is incomplete, and the rigidity depends on contraction of muscles or ligaments, we know that by extension and exercise we can overcome it: in true anchylosis, dependent on union of bone, remedial attempts would be worse than useless. In the application of machinery to the spine, the danger to be feared is injury to the spinal marrow; but such an accident cannot happen with common care.

"The employment of machinery to extend the spine, is the great secret of cure, in bad cases of curvature. The means used for this purpose are of less importance than prudence and care in their management. I prefer making extension with the patient in the horizontal position. The head and shoulders being fixed by proper pads and bandages to the head of the bed or sofa, extension is made by a screw or a pully at the foot, communicating with straps attached to the sides of a bandage which surrounds the pelvis. Having by a careful examination determined that the curvature is unconnected with disease of the cartilages or the bones, we must ascertain how far the vertebral column can be restored by extension." (P. 188.)

The earlier an attempt is made to cure deformity, the better; but there are few curvatures, originating in muscular derangements, which cannot be entirely effaced before the growth has ceased, and many old-established cases have been cured even some years after this period. In all the exercises employed, the muscles of both sides of the vertebræ should be equally exerted, as well as those of both arms. "A long pole held to the hands, with the arms fully extended, and moved in various directions, is a good modification of exercise; as is also turning a wheel by each hand at the same time."

The subjects next considered are diseases of the Fibro-cartilages and Vertebræ as causes of spinal distortion; and descriptions and diagrams are given of the mechanical apparatus used in the treatment of deformities.

If Mr. Beale cannot claim the merit of originality, he is at least entitled to our approbation for having made a useful compilation upon a very important class of diseases, which cannot fail to prove instructive to surgical students.

---

*An Experimental Inquiry into the Structure and Function of the Spleen.* By Wm. Dobson.—8vo. pp. 32. Wilson, 1830.

We shall not take a retrospective view of all the various opinions that have been entertained respecting the functions of the spleen; the task would be tedious to ourselves, and perfectly useless to our readers, as for the most part fancy has been brought into play, to supply a deficiency of facts. Dr. Dobson, by way of introduction, refers to the most plausible theories that have been advanced upon this subject. He just mentions the opinion of the ancients: they found the spleen contained a dark-coloured fluid, apparently resembling the bile, except in the blackness of its colour. This fluid they called "*atra bilis*;" and the use of the spleen they considered to be for the secretion of black bile. It was once believed that the spleen was a glandular organ, but no excretory duct has ever been found. The opinions of Dr. Haighton and Sir E. Home are deemed the most plausible: the former physiologist, finding the spleen diminished in size during a meal, considered that the distended stomach presses on the spleen, thereby determining a greater quantity of blood to the stomach, liver, and pancreas, during the digestive process. Sir E. Home's researches led him to conclude that the spleen consists of a congeries of blood-vessels and absorbents, without any connecting cellular membrane. From this mechanism Sir E. considers the *interstices of the vessels* to be a reservoir for the superabundant serum, lymph globules, soluble mucus, and colouring matter, carried into the circulation immediately after the process of digestion is completed. Sir Astley Cooper inferred, from his own investigations, that the spleen is an elastic reservoir and manufactory of venous blood. Mr. Hewson thought that the red globules of the blood were elaborated in this organ. Sir Anthony Carlisle regards "the compensating heat of the spleen to be the natural provision against the *torporizing* influence of low temperature suddenly applied to the nervous and muscular structures of the stomach." Mr. C. Bell regards the spleen as a provision for giving the vessels of the stomach an occasional power and greater activity, enabling them to pour out a quantity of fluid according to the necessity of digestion: and, in addition, he considers the venous blood of the spleen useful in aiding the function of the liver. But to proceed to the investigations of Dr. Dobson.

When experimenting on animals for the purpose of ascertaining the effects which different poisons produced on the animal economy, he was often struck with remarkable variations in the size of the spleen: on paying further attention to this incident, he observed that the spleen of a dog, killed a few hours after taking food, was of a very large size, when compared to the spleen of a dog that had fasted for several hours. On examining the different theories of the spleen, one remark was found which coincided to a certain extent with his own observation, that "the spleen is of a large size when the stomach is empty, and small when the stomach is full." Dr. Dobson now resolved to perform a series of experiments upon the subject. The first point of inquiry was to ascertain the changes produced in the spleen by the digestive process, and to ascertain the precise period at which any alteration should be manifest."

"Exp. I. I gave to a middle-sized dog a hearty meal of beef and mutton; the animal ate heartily. In four hours after, I opened the abdomen, and exposed the spleen immediately: it was large and firm; its veins appeared completely gorged with blood: on cutting into the organ, a large quantity of dark coloured blood flowed out: the exact amount could not be estimated; but I should suppose there was about four ounces: it concreted in a very short time. The coagulated mass, however, was soft, easily broken down, and presented more the appearance termed grumous blood, than the proper sanguineous fluid.

"Exp. II. A dog was procured as near in size as the one in the last experiment as could be met with; the animal took a full meal of beef and mutton; in five hours after, the abdomen was opened; the spleen was very large, and turgid with blood. The appearance of the blood was very similar to that in the last experiment; the quantity, however, was much greater.

"Exp. III. The spleen of a dog (of an equal size to the preceding) was examined twelve hours after any food had been taken: a very remarkable difference was observable; it was very small, and flabby, and contained only a very small quantity of blood. The appearance of the blood differed little from that in the preceding experiments: I thought it not quite so dark.

"To ascertain the comparative bulk of the spleen in dogs, I procured two of equal size, and examined their spleens at the same period after a meal; the difference in size was so trifling, as not to invalidate in the least the conclusions I intend to draw from the preceding statements." (P. 11.)

These experiments were repeated with similar results, and others also were performed at various periods, *during* the digestive process and *after* its completion. The size of the spleen was invariably found to be in a ratio to the quantity of nutriment taken into the system, and to the period at which it was examined after the animal had eaten: that is, to three hours after a meal, little alteration in this organ was perceptible; but in four hours after, it was large; and in about five hours appeared to arrive at its maximum, and then gradually to decrease in bulk for twelve hours, which was as far as its condition was observed.

Dr. Dobson next removed the spleen from a dog, to observe the effect which might be produced on the system: "the animal apparently suffered little from the operation." Next day it ate voraciously. For three hours no perceptible alteration was produced; but in *four hours after*, the animal became restless, and sunk into nearly a torpid state: there was every mark of plethora, or overfulness of the vascular system. In the course of two hours from this period the animal began to recover, and in three hours the symptoms had subsided; considerable languor remained. The animal took a large meal twice or thrice in twenty-four hours, and, after each, precisely similar effects were presented. It became more feeble daily, and died a month after the operation.

"I next removed the spleen from another dog, but instead of giving full meals, as in the last experiment, I gave a small quantity of food every hour, or every two hours. The animal ate voraciously; no unpleasant symptoms occurred. This plan was pursued for three weeks, when the animal to all appearance was quite well; in fact, it became fat: the ligature from the splenic artery had come away, and the wound in the abdomen healed. I then commenced giving full meals twice in twenty-four hours: the same train of symptoms followed each meal, and at the same period as in the last experiment, though perhaps not so urgent. The animal died in a month from the commencement of this plan of feeding." (P. 14.)

This experiment does not appear to us to have been well executed: the splenic artery should be tied with single threads cut close; the wound then heals at once. Our impression is, that the bad results of feeding with large meals in these instances was accidental, and independent of the removal of the spleen. It is not unlikely that the animals suffered severely from the seton (for such the ligature was,) kept in the abdomen three weeks, and perhaps died in consequence of this injury alone. Mr. Mayo (Outlines of Physiology,) kept one dog from which he removed the spleen: soon after it had recovered from the injury, it was freely fed, and yet it lived between three and four years in good health.

In both dogs the feces were of a lighter colour than natural.* On examining the body of each after death, a small quantity of limpid serum was contained in the bag of the tunica arachnoides, and more than a natural quantity in the lateral ventricles: the veins of the brain were in a highly congested state: abdominal viscera presented no unnatural appearances; but the portal system of veins was much gorged with blood.

*Corollaries regarding the functions of the spleen.* Dr. Dobson infers from his experiments, *that the spleen acts as a reservoir for containing the additional quantity of blood which the vascular system has received by means of the nutritive process.*

"It is evident from the remarks on chylification, there is a greater quantity of blood in the system at five hours after a meal than at any other period; and as we have premised that the blood-vessels are not capable of containing this increase with impunity, I infer that the spleen serves as a reservoir to hold this surplus; because at the time the chylifactive process is at an end, the spleen is found distended with blood. Then, as detailed in the third experiment, at twelve hours after a meal, the spleen was small, and contained very little blood: the reason of this phenomenon is obvious; at five hours after a meal, the nutritive process is nearly completed; at five hours after a meal, the spleen arrives at its maximum size: now, as secretion goes on in the various emunctories, there must consequently be a reduction of the circulating mass; and to compensate for this, blood is simultaneously expelled from the spleen, so that in twelve hours the whole is removed; no more circulating through that organ than is necessary for its support.

"We have now to examine the second series of experiments. When the spleen was removed from a dog, and full meals given to the animal, the effects indicated clearly that a greater quantity of blood had been formed than the vessels were capable of con-

* This was not the case in the dog whose spleen was removed by Mr. Mayo. —Editor.

taining, compatible with the free action of the vital organs; but as the fluid became diminished in quantity by the secretory functions, healthy action in these parts was again established. But it was observed, that if a small quantity of food was given at a time, though often repeated, no deleterious influence was exerted: that is, if the increase in the volume of blood was not more than equivalent to what had been previously expended by the secretions, no injurious effects were produced.

"I performed two experiments somewhat analogous to those which were made by Magendie.

"Exp. I. Ten ounces of blood were injected into the jugular vein of a dog, the abdomen being previously laid open. After the injection had proceeded for a short time, I observed the spleen to increase in size gradually until the whole of the blood was injected. This animal had fasted for ten hours, and previous to the operation the spleen was small.

"Exp. II. I opened the jugular vein of a dog five hours after eating, the abdomen being at the same time open: the spleen was large; as blood flowed from the vein, the spleen very perceptibly diminished in size, until the animal fainted. On grasping this organ at the time, a sensation was felt as if it was muscular, and was compressing out its contents with an active force. I did not perceive the slightest evidence of diminution of any other viscus.

"These experiments corroborate the deductions made from the former ones; they illustrate, in short, the principle which I am endeavouring to establish, that the circulatory vessels are capable of containing only a certain quantity of blood with impunity, and that when an increase in the volume is produced, as after digestion, the spleen performs the office of a reservoir to receive the surplus; they show also that, when the fluid contained in the vessels becomes reduced in quantity, as from bleeding, the spleen affords a supply, so as to enable the various organs to perform their necessary offices; and further, they afford collateral evidence of the spleen being more elastic than the blood-vessels." (P. 22.)

Some very brief but acute remarks are made on the pathology of the spleen.

The attempt Dr. Dobson has made to explain the function of this organ is very creditable to his ingenuity. It is more plausible than any other theory with which we are acquainted upon the subject, although we cannot venture to say that this essay completely succeeds in establishing it.

