# Erratum

**Published:** 1830-11

**Authors:** 


ERRATUM in our last Number, p. 342, two lines from top, for "have" read "has."

